# Effect of Olympic Weight Category on Performance in the Roundhouse Kick to the Head in Taekwondo

**DOI:** 10.2478/v10078-012-0004-x

**Published:** 2012-04-03

**Authors:** Isaac Estevan, Coral Falco, Octavio Álvarez, Javier Molina-García

**Affiliations:** 1 Department of Management and Applied Sciences in Physical Activity and Sport. Catholic University of Valencia, Spain.; 2 Department of Physical Activity and Sport Sciences. Catholic University of Valencia. Valencia, Spain.; 3 Department of Social Psychology. University of Valencia. Valencia, Spain.; 4 Department of Teaching of Musical,Visual and Corporal Expression, University of Valencia, Valencia, Spain.

**Keywords:** biomechanics, taekwondo combat, execution distance, body mass

## Abstract

In taekwondo, kick performance is generally measured using impact force and time. This study aimed to analyse performance in the roundhouse kick to the head according to execution distance between and within Olympic weight categories. The participants were 36 male athletes divided into three categories: featherweight (n = 10), welterweight (n = 15) and heavyweight (n = 11). Our results show that taekwondo athletes in all weight categories generate a similar relative impact force. However, the results indicate that weight has a large impact on kick performance, particularly in relation to total response time.

## Introduction

Taekwondo championships are organized according to weight categories. In the Olympic context, athletes are divided into four interval weight groups, from “flyweight” to “heavyweight”. In combat, taekwondo athletes aim to knock out or score more points than their opponent. To achieve this, athletes must be able to deliver sudden bursts of explosive and very powerful movements within a very short time period (Kazemi et al., 2009). One of the faster kicks that allows athletes to score more points and is the most used kind of kick in taekwondo is the roundhouse kick (Kim et al., 2010).

Despite the obvious importance of kick performance in combat sports, the best method of examining kick performance is not clear (Conkel et al., 1988; Gulledge and Dapena, 2008). The World Taekwondo Federation (WTF) competition rules establish that to score points, the kick must be powerful (WTF, 2010). In this context, Winter (1984) stated that force analysis (i.e., impact force) must be normalized according to body mass (i.e., relative impact force). On the other hand, from the points of view of technique and effectiveness, athletes must kick in the shortest time possible (Kazemi et al., 2009). Thus, kick performance must be measured by impact force, response time and also emulating the real situation (i.e., the weight category) (Waliko et al., 2005).

Weight categories in combat sports were established to normalize conditions in championships, enabling athletes to compete with opponents similar to themselves. However, only a few studies have considered weight category in analysing performance in Olympic combat sports (Atha et al., 1985); others even tried to consider different weight categories because athletes’ weight appears to affect performance (Waliko et al., 2005).

Although scientific knowledge about taekwondo is still relatively limited (Heller et al., 1998), research on mechanical analysis of combat sports has increased in recent years (Falco et al., 2009; Smith et al., 2000). Some authors who analysed kick performance found that, in general, it varies by execution distance (ED) (Bolander et al., 2009; Falco et al., 2009; Neto et al., 2007), although for athletes competing at a high level, the distance does not appear to compromise the efficiency of the action (Estevan et al., 2011). Likewise, kick performance also varies according to the athlete’s level and height (Conkel et al., 1998; Estevan et al., 2011; Falco et al., 2009; O’Sullivan et al., 2009), that is, kick performance is better among higher-level athletes and for lower kicks. However, no study has analysed kick performance in taekwondo in relation to the athlete’s weight.

The present paper is the first to analyse kick performance by taekwondo athletes taking into account each of these aspects. The aim of this study was to analyse the performance based on mechanical variables in the roundhouse kick to the head according to ED and between different weight categories.

## Material and Methods

### Subjects

The participants were 36 healthy male taekwondo athletes. Their average age was 25.03 ± 5.68 years, and their average body height was 1.78 ± 0.76 m. They were divided into groups according to the Olympic weight categories in competition. Three groups were created: featherweight (*n* = 10), between 58 and 68 kg (65.0 ± 2.8) (athletes’ level: two silver medalists in the world senior and police championships, five medalists in national senior and university championships and three medalists in the regional championship); welterweight (*n* = 15), between 68 and 80 kg (73.4 ± 3.7) (athletes’ level: seven medalists in national senior and university championships and eight medalists in the regional championship); and heavyweight (*n* = 11), over 80 kg (90.2 ± 5.6) (athletes’ level: eight medalists in national senior and university championships and three medalists in the regional championship). Every participant had at least four years of competition experience and trained for at least three hours per week. None of the athletes was colour-blind. All the participants signed an informed consent form and this study was approved by the relevant ethics committee of the university.

### Instruments

A new adaptation of the model created by Falco et al. (2009) was used in the present study to measure the mechanical variables. The adapted model allows measurement of longer time periods of the kick and a larger range of force with similar reliability to the original model. The data acquisition system was composed of a force platform and LEDs, both located in a dummy, an analogue-to-digital (A/D) microcontroller and a PC. The dummy allowed the force platform to be freely adjusted to the athlete’s body height. The force platform was built with nine piezoresistive pressure sensors (Flexiforce® A201 model by Tekscan Company Inc., South Boston, USA) positioned in series in groups of three in a triangular structure.

**Figure 1:**
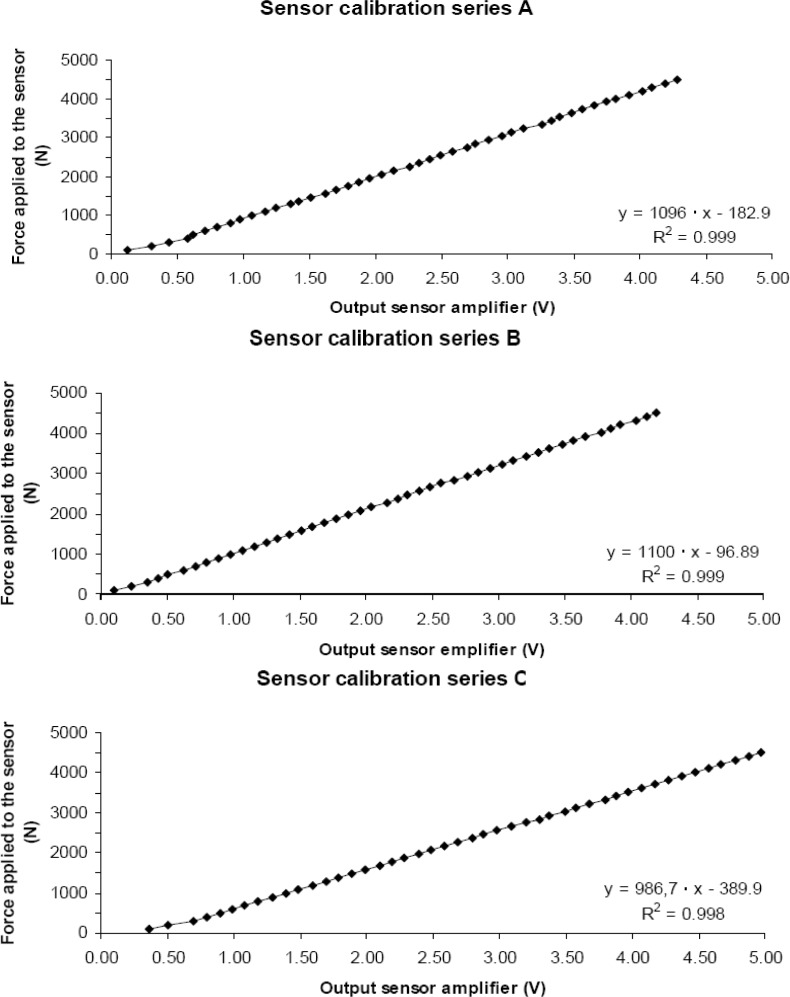
Example of the three series in the calibration sensor system.

The calibration of the force platform was carried out following recommendations by Falco et al. (2009) in a three-sensor by three-sensor series (Figure 1). To determine the actual force range that matched the sensor output range, a linear interpolation was done between zero load and the known calibration loads. We incrementally placed loads of 10 kg until we reached a total of 460 kg (almost 4508 N) to provide a constant drive voltage as well as an output voltage proportional to the applied force. The reliability of the system was measured by Cronbach’s alpha (Cronbach, 1951), which was 0.97. The mechanical variables were registered by a computer and Visual Basic 6.0 program was used to develop the software.

### Measurements

Three EDs and the kicking height (the athlete’s chin height) were determined for each athlete based on his anthropometric measures (leg length and chin height). Data were measured in May, two weeks after the European Senior and National University Taekwondo Championships. At this time, the athletes were training for the Spanish King Cup. The remainder of the protocol was executed according to Estevan et al. (2011). After a 20 min warm-up (each athlete was required to follow a general warm-up prior to testing by general mobility, 8 min jogging, 15 m sprint twice, kick 5–8 times, and stretching of the upper and lower limbs; they were also allowed a specific warm-up that consisted of performing six kicks as fast as possible and kick at least six times—twice per distance—to familiarize themselves with the process of kicking the dummy), the subject stood still in the guard position and waited for the blue signal. The onset position was characterized by a static disposition without the need for the heel of the back foot to rest on the floor. When the blue light signal changed to red, he kicked twice from each ED with the back foot (dominant lower limb; 29 right-footed and seven left-footed) in the indicated area of the dummy. The mean ED_1_ (short) was 0.68 ± 0.05 m, ED_2_ (normal) was 1.03 ± 0.07 m and ED_3_ (long) was 1.37 ± 0.09 m.

The total response time (TT) was the time from the visual signal to the instant when the kicking foot hit the target raising the maximum impact force or the sum of reaction and execution time (Pieter and Pieter, 1995). The impact force (IF) is defined as the maximum impact force in each kick. The relative impact force (RIF) is an estimation of the impact force according to the athlete’s body mass. These variables were analysed in the roundhouse kick to the head in taekwondo. This kick is defined as one in which the athlete puts the weight on the pivoting foot, while turning the body immediately folding the knee; as the knee stretches, the kicking foot makes a circle horizontally in order that the fore sole may kick the head or the target (Keum-Jae, 2005).

### Statistical analysis

Statistical analyses were performed using the SPSS 15.0 computer package. All parameters were normally distributed (Kolmogorov–Smirnov test). The intraclass correlation coefficient (ICC) for mechanical variables was determined. Values of mechanical variables were normalized (z-score) to compare data between groups according to the Olympic category. A mixed ANOVA model was used to compare mechanical variables between execution distances in the same Olympic category; pairwise comparisons were performed using Bonferroni statistics. Cohen’s *d* score was quantified to analyse the effect size (Cohen, 1988). The statistical significance was set at *p* < 0.05.

## Results

Table 1 sets out the statistical descriptions of all mechanical variables (TT, IF and RIF) for each group for the three EDs. The TT had an ICC R = 0.63 (95% IC, 0.42–0.78), the IF had an ICC of R = 0.86 (95% IC, 0.78–0.91), and the RIF had an ICC of R = 0.77 (95% IC, 0.64–0.86).

*Comparisons between groups:* The mixed ANOVA model using normalized data (z-score) showed that from ED_2_, the heavyweight group kicked with larger IF than the featherweight group (*p* < 0.03); the normalized effect size *d* was 1.49. In addition, from ED_1_ and ED_2_, the heavyweight group kicked in a longer TT than the welterweight group (*p* < 0.05) and (*p* < 0.02), with *d* values of 1.13 and 1.12, respectively. Finally, from ED_1_, the heavyweight group kicked in a longer TT than the featherweight group (*p* < 0.03), and *d* was 1.18. No differences in RIF were found from any distance.

*Comparisons within groups:* For the featherweight group, the mixed ANOVA model using Bonferroni statistics adjusted for paired comparisons showed a longer TT from ED_3_ than ED_1_ and ED_2_, and a longer TT from ED_2_ than ED_1_ (*p* < 0.001); the value of *d* was 1.16, 1.94 and 3.33, respectively. Finally, the IF (*p* < 0.04) and RIF (*p* < 0.03) were larger in kicks made from ED_1_ than ED_3_; the *d* value was 0.93 and 1.01, respectively.

For the welterweight group, the TT was longer from ED_3_ than ED_1_ or ED_2_ (*p* < 0.001), and longer from ED_2_ than ED_1_ (*p* < 0.01); the *d* value was 2.20, 1.69 and 0.67, respectively.

For the heavyweight group, the TT was longer from ED_3_ than ED_1_ (*p* < 0.001) and ED_2_ (*p* < 0.03), and the *d* value was 2.56 and 1.85, respectively. Finally, the IF and RIF were larger in kicks made from ED_2_ than from ED_3_ (*p* < 0.03); the *d* value was 0.59 and 0.64, respectively.

## Discussion

The purpose of this study was to analyze mechanical variables such as total response time and impact force in the roundhouse kick to the head according to the execution distance among different Olympic weight categories. To that end, a data acquisition system was adapted from Falco et al. (2009). This adapted model allowed the measurement of longer periods of kick performance (i.e., total response time) in taekwondo. This system advances in line with Heller et al. (1998), who stated that laboratory testing should not differ from the specific conditions of a taekwondo competition.

The results of this study show that weight has a strong effect on kick performance, particularly in relation to total response time. That is, athletes in the heavyweight group kick with a longer total response time than those in featherweight and welterweight groups. However, taekwondo athletes from all groups generate a similar IF per kilogram of body mass (i.e., relative impact force), as well as a similar IF (except from the normal distance, where heavyweights kick with higher IFs). In training, coaches are not concerned if athletes from different weight categories work together in pairs, because athletes in lower weight categories kick with a force similar to that of athletes in higher weight categories. Indeed, coaches can take advantage of this situation to improve the TT of heavyweight athletes. Nonetheless, it is appropriate for championships in combat sports to divide participating athletes according into their weight because this creates a more homogeneous context; we could therefore state that athletes are in initially similar performance conditions.

The present findings indicate that ED also affects the kick performance (Estevan et al., 2011; Falco et al., 2009) when participants are divided into Olympic weight categories, particularly in relation to time performance. This is in contrast to Neto et al. (2009), who reported that time performance may be less affected than force in certain conditions. In our study, we found longer response time from longer distances (normal and long) in every weight category; however, only IF was lower from a long distance in the featherweight and heavyweight groups. It appears that in taekwondo, time (i.e., TT) is affected more by ED than by force variables, with such time differences being large. Our results suggest that the ED has less effect on impact force when athletes are divided into level groups (Estevan et al., 2011; Falco et al., 2009; Neto et al., 2009). Therefore, taekwondo coaches should focus their training on improving time performance from different EDs, because this is linked to successful kick performance (Kazemi et al., 2009).

Regarding the IF according to ED, feather and heavy athletes kick with a lower IF from a long ED, and welters kick with a similar IF from every ED. This is in contrast to upper limb strikes in combat sports, in which the IF increases with longer EDs (Bolander et al., 2009; Gulledge and Dapena, 2008; Neto et al., 2007). Bolander et al. (2009) stated that larger IFs are developed from longer EDs from the opponent, because of an increase in the time available to accelerate the upper limb strikes. Future studies should analyse the execution (i.e., using motion capture systems) in which kick technique is based on, because the finding might lead to understanding of the mechanism of the kick in combat sports. This procedure would enable analysis of the important factors in combat sports that Sadowski (2005) pointed out, such as motor adjustments and reaction to unexpected signals.

A limitation of the present study is that there were no participants from the flyweight category (less than 58 kg). Hence, our results have a limited external validity to the other three Olympic male categories (featherweight, welterweight and heavyweight). Along similar lines, it would also be necessary to analyse the kick performance between female weight categories in order to improve technique and performance knowledge among female athletes.

## Conclusion

This study showed that weight category and execution distance affect kick performance in male taekwondo athletes. In taekwondo conditions that are more real (such as athletes divided into weight category), coaches must focus their training programmes to improve time performance, because it is affected by the execution distance in all weight categories.

## Figures and Tables

**Table 1:** Comparative analysis of performance by taekwondo athletes from different Olympic weight categories according to execution distance

	ED	Featherweight group (*n* =10)		Welterweight group (*n* =15)		Heavyweight group (*n*=11)	
IF (N)	ED_1_	1156.50 ±395.95	a	1237.47±367.99		1402.14±642.80	a
	ED_2_	930.15±279.00	a	1174.50±510.76		1464.41±448.93^*^	a
	ED_3_	849.90±288.36	b	1102.07±404.35		1148.77±647.11	b
RIF (N·kg^−1^)	ED_1_	17.60±5.42	a	16.96±4.54		15.91±5.98	
	ED_2_	14.20±3.80	a	15.62±6.40		16.78±4.11	
	ED_3_	13.01±4.12	b	15.01±5.22		13.29±6.99	
TT (s)	ED_1_	0.767±0.07	a	0.764±0.07	a	0.848±0.08^*†^	a
	ED_2_	0.850±0.08	b	0.816±0.07	b	0.901±0.09	a
	ED_3_	0.988±0.07	c	0.986±0.13	c	1.069±0.10^†^	b

ED = execution distance; IF = impact force; RIF = relative impact force; TT = total response time. Level of significance of difference is set at p < 0.05. A different letter (a, b, c) to the right of the mean ±SD indicates significant differences between distances in this group.

* Significant difference between featherweight and heavyweight group.

† Significant difference between welterweight and heavyweight group.
